# Seeing the past through rose-colored glasses? Age differences in recounting a difficult memory

**DOI:** 10.1093/geroni/igaa057.3265

**Published:** 2020-12-16

**Authors:** Arya Jones, Stephanie Wilson, M Rosie Shrout, Janice Kiecolt-Glaser

**Affiliations:** 1 Rice University, Southlake, Texas, United States; 2 Southern Methodist University, Dallas, Texas, United States; 3 Institute for Behavioral Medicine Research, Columbus, Ohio, United States; 4 The Ohio State University College Of Medicine, columbus, Ohio, United States

## Abstract

According to socioemotional aging theories, people better regulate their emotions in older age by reframing stressors and focusing on the positive aspects of difficult experiences. However, empirical results have been mixed. To address this gap, we examined age differences in the language use and cardiovascular reactivity of 188 adults (mean age=56, range=40-86) who relived an upsetting memory from their past. Consistent with theory, results revealed that older adults used significantly fewer negative emotion words and, among the negative emotions, marginally fewer words of anger, to describe their upsetting memory. Notably, however, there were no age differences in the expression of positive emotion or sadness. Controlling for education and cognitive function, greater expression of anger was associated with heightened systolic blood pressure (SBP) reactivity among older adults, not middle-aged individuals. Despite their expression of less negative emotion, older adults’ heart rate variability (HRV) dipped lower during disclosure than did middle-aged adults’. However, among those who used more positive emotion, sadness, and/or cognitive processing words, older adults no longer showed lower HRV than middle-aged participants. Overall, these results provide some evidence of positivity bias among older adults even when asked to recount a distressing personal memory, although this trend was not consistent for the expression of sadness or positive emotion. Further, cardiovascular responses appear more clearly tied to older adults’ level of engagement and emotional focus compared to their middle-aged counterparts’.

